# Artroplastia na osteoartrite do polegar (articulação carpometacarpal): Evitando a trapezectomia

**DOI:** 10.1055/s-0045-1812998

**Published:** 2025-12-15

**Authors:** Marcio Aurelio Aita, Giulia Cordeiro Aita, Cleyton Rocha, Sullivan Savaris, Mauricio Leite, Samuel Pajares Cabanilla

**Affiliations:** 1 Divisão de Ortopedia, Departamento de Cirurgia, Faculdade de Medicina do ABC, Santo André, SP, Brasil; 2 Curso de Medicina, Escola de Saúde, Universidade Municipal de São Caetano do Sul (USCS), São Caetano do Sul, SP, Brasil; 3 Departamento de Ortopedia, Ortoclin, Camboriú, SC, Brasil; 4 Divisão de Ortopedia, SOS Mão, Recife, PE, Brasil; 5 Policlínica Gipuzkoa, San Sebastián, Espanha

**Keywords:** articulações carpometacarpais, artroplastia, osteoartrite, polegar, trapézio, arthroplasty, carpometacarpal joints, osteoarthritis, thumb, trapezium bone

## Abstract

**Objetivo:**

Mostrar os resultados clínicos pós-operatórios, incluindo o tempo de retorno às atividades da vida diária (AVDs) relatado pelo paciente, os aspectos radiográficos e a força de preensão, da artroplastia total em pacientes com rizartrose em estágio III.

**Métodos:**

Neste estudo prospectivo e unicêntrico, analisou-se a artroplastia total com prótese não cimentada de metal-polietileno (Maia, Groupe Lepine). Os critérios de inclusão foram pacientes com osteoartrite da articulação carpometacarpal (CMC) em estágio III, idade acima de 60 anos, tratados inicialmente com procedimentos não cirúrgicos. Os critérios de exclusão foram pacientes com doenças mentais, abuso de álcool e artrite reumatológica.

**Resultados:**

Durante o estudo (de janeiro de 2018 a outubro de 2023), 34 pacientes atenderam aos critérios de seleção. Três pacientes elegíveis receberam outro implante, e dois foram simultaneamente submetidos a cirurgia em outra articulação (metacarpofalangiana com deformidade em Z); estes indivíduos não foram incluídos no estudo. Após 25,1 meses da cirurgia, a força de preensão era de 87,75% em relação ao lado oposto, as pontuações na Escala Visual Analógica (EVA) e na versão curta do questionário de Incapacidade do Braço, Ombro e Mão (Quick Disabilities of the Arm, Shoulder and Hand, QuickDASH) eram de 1, e a amplitude de movimento era de 81% em comparação ao lado não acometido. No exame radiográfico, observou-se subsidência inicial do metacarpo em 100% dos pacientes. Dois pacientes (6,9%) apresentaram complicações.

**Conclusão:**

A escolha da artroplastia total para o tratamento de pacientes ativos com mais de 60 anos e artrite da articulação CMC em estágio III preserva a independência para a realização de AVDs e melhora a qualidade de vida nos primeiros 12 meses após o procedimento.

## Abstract (en)

**Objective:**

To show the postoperative clinical results, including the patient-reported time to return to the activities of daily living (ADLs), the radiographic aspects, and the grip strength of total joint arthroplasty in patients with stage-III rizarthrosis.

**Methods:**

In the present single-center and prospective study, we analyzed total joint arthroplasty with an uncemented, metal-on-polyethylene prosthesis (Maia, Groupe Lepine). The inclusion criteria were patients with stage-III carpometacarpal (CMC) joint osteoarthritis, aged > 60 years, initially treated through non-surgical procedures. The exclusion criteria were patients presenting mental illness, alcohol abuse, rheumatological arthritis.

**Results:**

During the study period (from January 2018–October 2023), 34 patients met the patient selection criteria. Three eligible patients received a different implant, and two were simultaneously submitted to surgery in another joint (metacarpophalangeal joint with Z-deformity); these subjects were not included in the study. After 25.1 months of the surgery, grip strength was of 87.75% regarding the opposite side, the scores on the Visual Analogue Scale (VAS) and on the short form of the Disabilities of the Arm, Shoulder, and Hand (QuickDASH) questionnaire were of 1, and the range of motion was of 81% compared to the unaffected side. On the X-ray examination, initial metacarpal subsidence was observed in 100% of the patients. Complications were observed in 2 (6.9%) patients

**Conclusion:**

The choice of total joint arthroplasty to treat active patients aged > 60 years with stage-III CMC joint arthritis preserves independence to perform ADLs and improves quality of life in the first 12 months of the procedure.

## Introdução

A prevalência da artrite basilar do polegar aumenta com a idade e reduz a capacidade de realização de atividades da vida diárias (AVDs). 1 É provável que o número de pacientes que procuram tratamento para esta doença tenda a crescer. 2 Porém, não há consenso quanto à prevenção da trapeziectomia total na artrite em estágio III, segundo a classificação de Eaton.

Há dúvidas sobre o aumento de complicações e custos devido à complexidade da cirurgia. 2 A artroplastia total na osteoartrite trapeziometacarpal pode ser um procedimento seguro em pacientes com mais de 60 anos e em estágio III após a falha do primeiro tratamento não cirúrgico. 3 4 5 6

Nesses casos, a artroplastia total é conveniente, não impede a realização de outras técnicas em caso de falha e é um método reprodutível que evita a rigidez, mantém o comprimento do polegar, melhora a amplitude de movimento inicial, permite a recuperação mais rápida e é uma alternativa à trapezectomia total. 7

O objetivo deste estudo foi apresentar os resultados clínicos pós-operatórios da artroplastia total, incluindo o tempo de retorno das AVDs relatado pelos pacientes, os aspectos radiográficos e a força de preensão manual 2 em pacientes com rizartrose em estágio III.

### Maior Custo

A necessidade de implante (prótese Maia, Groupe Lepine) aumenta o custo do tratamento e faz com que os planos de saúde relutem em oferecer cobertura aos pacientes, apesar das evidências crescentes de seu benefício.

## Métodos

O estudo obedeceu aos padrões éticos e foi aprovado pelo Comitê de Ética em Experimentação Humana. Um termo de consentimento livre e esclarecido foi fornecido a todos os participantes da pesquisa, que o leram e assinaram de acordo com sua vontade.

Este estudo prospectivo e unicêntrico analisou a artroplastia total com prótese não cimentada de metal-polietileno (Maia, Groupe Lepine). O Comitê de Ética Institucional aprovou o termo de consentimento livre e esclarecido por escrito e os pacientes o assinaram antes da inclusão no estudo. O critério de inclusão foi pacientes com osteoartrite da articulação carpometacarpal (CMC) em estágio III e idade superior a 60 anos, inicialmente tratados com procedimentos não cirúrgicos. Os critérios de exclusão foram pacientes com doenças mentais, abuso de álcool e artrite reumatológica.

Durante o estudo (de janeiro de 2018 a outubro de 2023), 34 pacientes atenderam aos critérios de seleção. Três pacientes elegíveis receberam outro implante e dois foram simultaneamente submetidos à cirurgia em outra articulação (metacarpofalangiana com deformidade em Z); estes indivíduos não foram incluídos no estudo.

### Análise Post hoc

A análise post hoc demonstrou que 29 dos pacientes incluídos no estudo não apresentaram diferenças em relação à idade, sexo ou estágio da artrite da articulação CMC com um poder estatístico de 85% em teste bilateral e nível de significância de 5%. 8 9 A amostra incluiu 25 pacientes do sexo feminino e 4 do sexo masculino com idade média de 64 anos (faixa: 60–74). A rizartrose foi classificada de acordo com o sistema de Eaton. 10 Todos os pacientes estavam disponíveis após 25,1 (faixa: 12–66) meses de acompanhamento.

### Técnica Cirúrgica

**Vídeo 1** Fluoroscopia dinâmica do polegar: a artroplastia total permite o restauro do comprimento do polegar e do equilíbrio do tendão, a estabilização da base do polegar e o aumento da abdução do polegar.


O objetivo do tratamento é melhorar o equilíbrio entre a mobilidade e a estabilidade da articulação CMC. A artroplastia total deve estar perfeitamente posicionada/fixada para permitir a osseointegração. 7

**Fig. 1:**
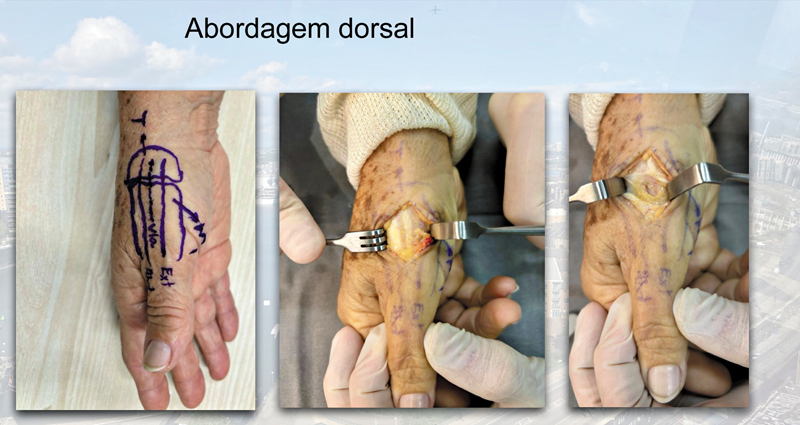
Abordagem dorsal do polegar entre o abdutor longo do polegar e os tendões extensores curtos.

**Fig. 2:**
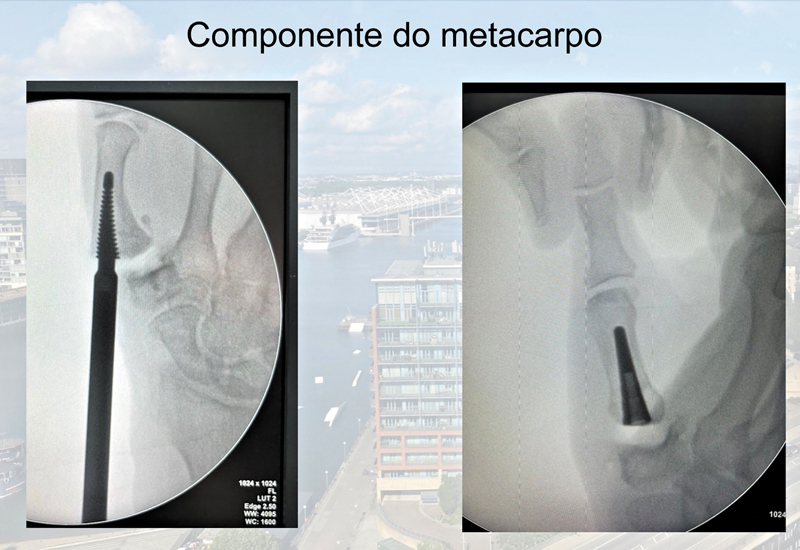
Componente do metacarpo.

**Fig. 3:**
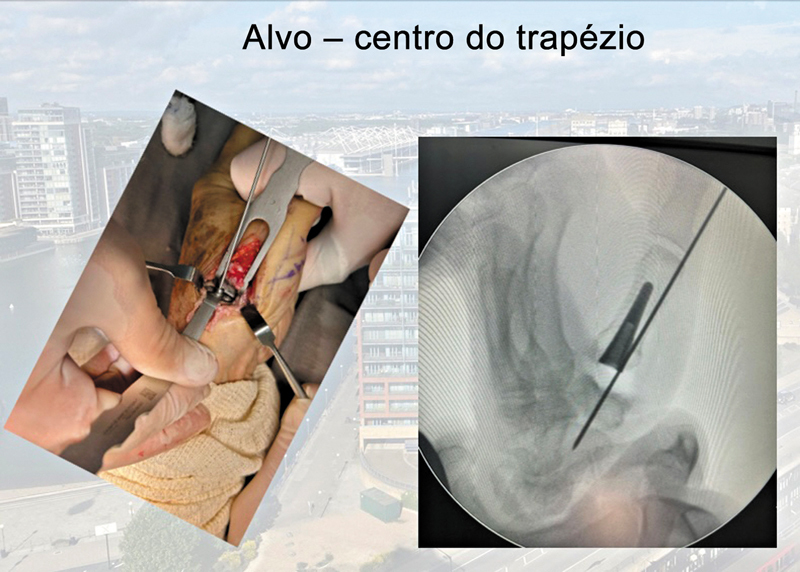
Componente do trapézio: centro de movimento da articulação carpometacarpal (realizado sob fluoroscopia).

A abordagem dorsal foi escolhida ( Fig. 1 ). A primeira etapa foi remover a superfície articular da base do polegar, incluindo os osteófitos volares e do bico medial. Em seguida, preparamos o canal medular metacarpal com brocas de manobra específicas de tamanho crescente, até obter estabilidade de encaixe por pressão e alinhamento da haste do eixo. O implante final foi inserido e nivelado à base do metacarpo. ( Fig. 2 ). A seguir, realizamos a colocação da cúpula no trapézio, com cuidado para evitar a fixação mecânica da cúpula não cimentada com o osso subcondral central e a superfície articular distal do trapézio. A cúpula precisa estar perfeitamente centralizada no trapézio e no centro de movimento da articulação CMC. A melhor direção para a passagem do fio-guia no centro do trapézio é 30° radiais entre o eixo longitudinal das diáfises do primeiro e segundo metacarpos (plano coronal) e o eixo anterior (plano sagital), com auxílio de fluoroscopia ( Figs. 3 4 ). A ressecção parcial da cápsula articular, com remoção dos corpos livres e preservação dos ligamentos palmares, foi feita. Há necessidade de equilíbrio entre os tecidos moles e o implante para melhorar a estabilização e evitar a rigidez. A avaliação do comprimento da articulação CMC do polegar pode ocorrer pela comparação do comprimento do primeiro e do segundo raios antes e após o implante dos componentes. Esta avaliação também pode ser feita por fluoroscopia, com análise da congruência do primeiro arco metacarpal em vistas anteroposteriores com o polegar em abdução de 45°, como o “arco gótico” ( Fig. 5 ).

**Fig. 4:**
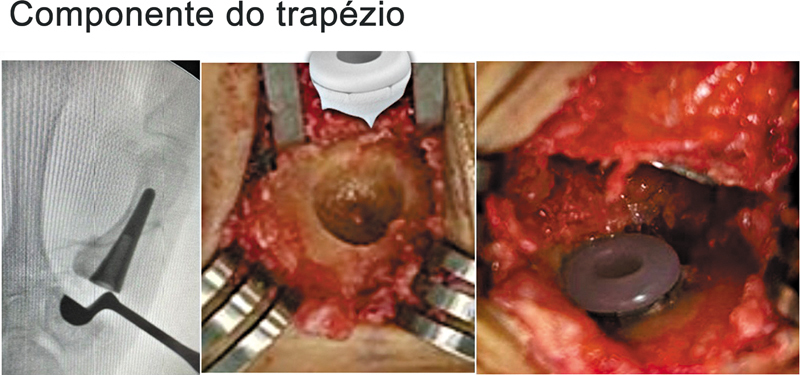
Componente do trapézio: a cúpula precisa estar bem centralizada e fixada ( *press fit* ) no trapézio.

**Fig. 5:**
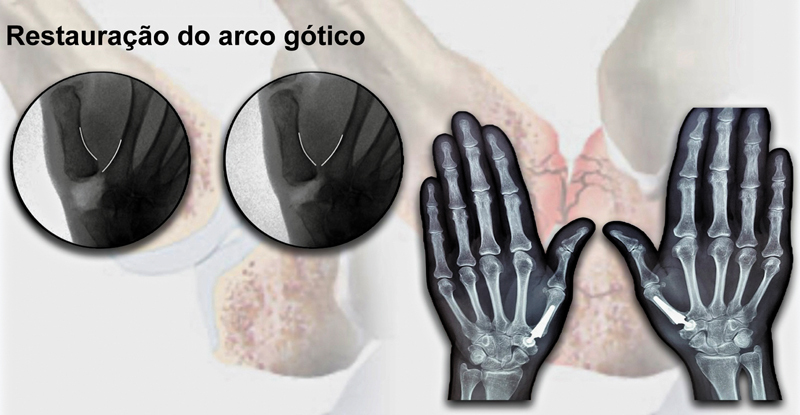
“Arco gótico”: congruência do primeiro e segundo arco metacarpal na vista anteroposterior com o polegar em 45° de abdução.

A artroplastia total permite o restauro do comprimento ( Vídeo 1 ) e do equilíbrio do tendão, a estabilização da articulação CMC e o aumento da amplitude de movimento do polegar. 7

### Cuidado Pós-operatório

Uma tala longa para o polegar foi aplicada ao final da cirurgia e mantida até 2 semanas após o procedimento. As suturas cutâneas foram removidas para que os pacientes pudessem movimentar ativamente o polegar. Uma imobilização gessada curta e removível foi colocada no polegar para uso noturno e durante as AVDs. O movimento de pinça deve ser estimulado após 3 semanas e, a seguir, os pacientes não têm mais restrições.

Todos os pacientes foram avaliados radiográfica e clinicamente às 6 semanas e novamente aos 6 e 12 meses após o procedimento. 11

A amplitude de movimento (AdM) foi avaliada com um único instrumento (goniômetro).

A força de preensão foi medida com dinamômetro de mão Jamar específico (Sammons Preston) e o valor obtido foi expresso como uma porcentagem daquele apresentado no lado oposto. 12 13 Estes valores foram classificados em quatro grupos de acordo com os quartis de força de preensão em relação ao tempo de retorno às AVDs. 14

Os desfechos clínicos foram aferidos pela Escala Visual Analógica (EVA) de dor (que varia de 1 a 10, em que 1 indica ausência de dor). A qualidade de vida foi determinada pelo questionário rápido *Disabilities of the Arm, Shoulder and Hand* (QuickDASH) (com pontuação de 0 a 100, em que 1 indica o melhor resultado). 15 16

A taxa de complicações foi avaliada nos mesmos tempos descritos.

### Parâmetros Radiográficos

Os parâmetros principais foram subluxação dorsal, aspecto dos implantes e subsidência metacarpal (para manutenção do espaço articular entre o trapézio e o metacarpo), que foram avaliados em 6 semanas, 6 meses e 12 meses após a cirurgia.

### Métodos Estatísticos

A Tabela 1 mostra as características demográficas basais e os detalhes das lesões encontradas na amostra. Os dados foram apresentados como média ou mediana de acordo com o tipo de variável e sua distribuição. A Tabela 2 revela os resultados objetivos e relatados pelos pacientes. A pontuação média da EVA e do QuickDASH foi de 1 ponto. A AdM foi de 97,15% em comparação ao lado não acometido. Ao exame radiográfico, para preservação dos parâmetros iniciais (implantes sem luxação e sem falha), observou-se subsidência metacarpal em 100% dos pacientes. Dois (6,9%) pacientes apresentaram complicações. Uma delas foi uma fratura do trapézio durante a cirurgia, tratada com fixação com fio de Kirschner e consolidação óssea. Outro paciente apresentou dor e diminuição da abdução do polegar, sendo tratado com exercícios de reabilitação, mas com manutenção da EVA em 3.

**Tabela 1:** Dados demográficos da amostra, lado das lesões e doenças associadas

Identificação	Idade	Profissão	Lado	Sexo	Doenças associadas
1	69	Cozinheira	Direito	Feminino	—
2	63	Atendente	Direito	Feminino	—
3	64	Estilista	Direito	Feminino	—
4	63	Estilista	Esquerdo	Feminino	—
5	61	Secretária	Esquerdo	Feminino	—
6	60	Professora	Esquerdo	Feminino	—
7	63	Professora	Direito	Feminino	—
8	60	Empresária	Direito	Feminino	—
9	66	Contadora	Esquerdo	Feminino	—
10	62	Secretária	Esquerdo	Feminino	—
11	66	Enfermeira	Esquerdo	Feminino	—
12	60	Vendedora	Direito	Feminino	STC
13	63	Costureira	Direito	Feminino	—
14	70	Juíza	Esquerdo	Feminino	—
15	64	Empresária	Esquerdo	Feminino	STC
16	67	Doméstica	Direito	Feminino	—
17	62	Doméstica	Direito	Feminino	Tenossinovite de De Quervain
18	63	Cozinheira	Direito	Feminino	—
19	67	Contadora	Direito	Feminino	—
20	70	Juiz	Direito	Masculino	Tenossinovite de De Quervain
21	64	Confeiteira	Direito	Feminino	—
22	60	Motorista	Direito	Masculino	—
23	62	Vendedora	Direito	Feminino	Gânglio
24	60	Empresária	Esquerdo	Feminino	—
25	70	Juiz	Esquerdo	Masculino	STC
26	64	Doméstica	Esquerdo	Feminino	Tenossinovite de De Quervain
27	63	Designer	Esquerdo	Feminino	Artrite IFD
28	66	Doméstica	Direito	Feminino	STC
29	62	Empresário	Direito	Masculino	—

**Abreviações** : IFC, interfalangeana distal; STC, síndrome do túnel do carpo.

**Tabela 2:** Resultados objetivos e relatados pelos pacientes

Identificação	Acompanhamento (meses)	AdM (% do lado oposto) aos 12 meses	QuickDASH	EVA	Força de preensão (% do lado oposto)	Retorno às AVDs (meses)	Complicações
1	39	81	1	1	96	1	—
2	40	90	1	1	97	2	—
3	34	84	1	1	94	1	—
4	34	88	1	1	96	1	—
5	31	91	1	1	94	2	—
6	31	90	1	1	94	2	—
7	29	82	1	1	98	1	—
8	29	78	1	1	97	2	—
9	20	82	1	1	99	1	—
10	66	88	1	2	83	5	Fratura de trapézio
11	76	91	1	1	91	1	—
12	36	80	1	1	96	1	—
13	14	78	1	1	92	1	—
14	12	74	1	1	94	2	—
15	17	79	1	1	96	1	—
16	16	82	1	1	98	1	—
17	18	83	1	1	87	2	—
18	19	91	1	1	98	2	—
19	14	90	1	2	96	1	—
20	14	90	1	1	96	1	—
21	14	82	5	3	87	6	—
22	24	88	1	1	96	1	—
23	21	73	1	1	91	1	—
24	20	89	1	2	83	1	—
25	12	70	11	2	82	5	Dor
26	12	64	1	1	81	1	—
27	12	63	1	1	96	2	—
28	12	66	1	1	92	1	—
29	12	62	1	1	97	1	—
**Média**	**25,10**	**81**	**1**	**1**	**87,75**	**2**	

**Abreviações** : AdM, amplitude de movimento; AVDs, atividades da vida diária; DASH, *Disabilities of the Arm, Shoulder and Hand* ; EVA, escala visual analógica.

## Discussão

Nos últimos anos, vários estudos consideraram os resultados de múltiplas técnicas de trapeziectomia total com suspensoplastia como bem-sucedidos. No entanto, novas pesquisas mostraram-se infrutíferas e sugeriram outros procedimentos (que não impeçam a realização de outras técnicas em caso de falha) para permitir uma recuperação mais rápida, com menos dor e maior força do polegar, como a artroscopia/sutura com botão ou a artroplastia total. 1 2 11

A força de preensão é um método válido e confiável para análise de desfechos objetivos e é um fator preditivo independente de incapacidade de realização de AVDs pelos pacientes. Forças de preensão nos quartis menores (primeiro ou segundo) aumentam o risco de incapacidade de realização de AVDs em comparação a forças nos quartis maiores (terceiro ou quarto). 13 14

Todos os pacientes deste estudo apresentaram valores no quarto quartil.

Bricout and Rezzouk relataram uma taxa de falha de 7,7% na implementação da prótese Maia em uma série de 156 pacientes. 17 Maeda et al. 11 mostraram uma taxa de complicações de 9,3%. Observamos menor incidência de complicações (6,9%) e os desfechos clínicos apresentaram maior AdM, tempo mais curto de retorno às AVDs e escore menor na EVA do que em outros estudos. 2 6 7

Uma crítica à redução da dor, complicações e custos da trapezectomia total tradicional é clara, mas, hoje, o pilar do tratamento é manter o comprimento do trapézio e do metacarpo e reduzir a subluxação dorsal do metacarpo do polegar 18 para aumentar a força de preensão e respeitar o conceito de não impedir a realização de outras técnicas em caso de falha (conceito “ *no burnt bridges* ”). Newton e Talwalkar, 2 Duerinckx e Verstreken 7 e este estudo demonstraram que a artroplastia total tem certas vantagens sobre outras opções, incluindo estabilização e alinhamento da articulação CMC e preservação da capacidade de executar as AVDs e do comprimento do polegar. Hustedt et al. 6 mostraram que o tempo de retorno ao trabalho foi de cerca de 4,5 meses em pacientes submetidos à trapezectomia total. Em nosso estudo, 93,1% dos pacientes submetidos ao procedimento retornaram ao trabalho antes de 2 meses.

Farkash et al. 19 mostraram uma taxa de falha de 11,32% em pacientes tratados com artroplastia parcial da articulação CMC. As desvantagens desse método incluem os custos, a dificuldade técnica da cirurgia e a possível taxa de complicações.

Hoje, o conceito de “no burnt bridges” é aceito, e a cirurgia definitiva precoce para tratamento de artrite da articulação CMC, por exemplo, trapezectomia total, é controversa, pois esgota as possibilidades terapêuticas cirúrgicas. Esse conceito recomenda o uso de métodos de artroscopia ou artroplastia do polegar 20 21 sempre que possível. Tal abordagem melhora a qualidade de vida dos pacientes e reduz o tempo de retorno às AVDs e às atividades laborais. Além disso, em caso de falha, é possível realizar todos os métodos cirúrgicos de revisão.

Este estudo é uma pesquisa clínica prospectiva, e todos os pacientes foram operados por apenas um cirurgião de mão, constituindo um grupo uniforme com acompanhamento completo. No entanto, algumas limitações precisam ser reconhecidas. Este estudo é uma série de casos e não um estudo clínico randomizado, o critério de inclusão foi o estágio III, de acordo com a classificação de Eaton, e o tamanho da amostra foi pequeno para análise do QuickDASH, da EVA e da força de preensão.

O aumento do custo é a principal razão para a dificuldade de realização da artroplastia total em pacientes que dependem de planos de saúde. O método e o implante para tratamento da artrite do polegar evoluíram exponencialmente, gerando desfechos excelentes, e são uma opção antes que estudos comparativos possam considerá-los o padrão-ouro e o procedimento de escolha. 21

## Conclusão

A artroplastia total para tratamento de pacientes ativos com mais de 60 anos e artrite da articulação CMC em estágio III preserva a independência para realização de AVDs e melhora a qualidade de vida nos primeiros 12 meses após o procedimento.
